# Health and nutrition emergency response among internally displaced persons at Ranch collective site, Chagni, Ethiopia: The role of emergency operation center, lessons from the field, and way forwards

**DOI:** 10.3389/fpubh.2022.926551

**Published:** 2022-09-15

**Authors:** Tefera Alemu, Belay Bezabih, Abraham Amsalu, Eyaya Hassen, Mahteme Haile, Melkamu Abite

**Affiliations:** ^1^ICAP in Ethiopia, Amhara Regional Office, Bahir Dar, Ethiopia; ^2^Amhara NRS Public Health Institute, Bahir Dar, Ethiopia; ^3^Awi Zonal Health Department, Injibara, Ethiopia; ^4^The House of Peoples' Representatives of FDRE, Addis Ababa, Ethiopia; ^5^Amhara Regional Health Bureau, Bahir Dar, Ethiopia

**Keywords:** emergency response, IDPs, health, nutrition, PHEOC, Ranch collective site

## Abstract

**Background:**

In October 2020, about 79,041 ethnically Amhara/Agew people had been internally displaced (IDPs) from Metekel zone of Benishangul-Gumuz region and lived in Ranch collective site, Chagni town, Ethiopia. Onsite PHEOC met the health and nutrition needs of the IDPs as per international humanitarian response standards.

**Methods:**

On January 11/2021, the Amhara Public Health Institute (APHI) established an onsite Public Health Emergency Operation Center (PHEOC) at Ranch collective site. Health workers and vehicles were deployed. A temporary clinic having nine outlets was built. Drugs and medical supplies were mobilized from different sources. The overall response period lasted about 8 months, from December 2020 up to June 2021.

**Results:**

A total of 33,410 IDPs had received free essential health services. Mental health and psychosocial support services had been given for 1,803 cases. Specialized medical services such as trachomatous trichiasis (30), cataract surgery (8) and sputum samples for mycobacterium tuberculosis (120) have been done. Moreover, 454 women received antenatal care services and 137 women gave birth at health facilities. About 837 children have got measles supplementary dose and 1,280 adults took a COVID-19 vaccination. A total of 1,448 children under five, 454 pregnant and 402 lactating women had been screened on monthly basis. Of which, severe and moderate malnutrition rate was 46 (3.2%) and 75 (5.2%), respectively. A total of 194 trench latrine seats, 74 shower rooms and 50 hand washing facilities had been constructed. There were no human feces present nor solid wastes accumulated around the shelters or settlements. Both active and passive surveillance activities were carried out throughout the camp life. We also conducted regularly Risk Communication and Community Engagement activities on priority health issues.

**Conclusion:**

We adequately met the health and nutrition needs of the IDPs as stated in the Sphere humanitarian handbook. We sought to have a strong Incident Management System and coordination platforms like PHEOC, a resilient health system, a training curriculum called Leading in Emergencies, and a multipurpose collective center with infrastructures, humanitarian response guidelines, training materials, and risk/vulnerability-based preparedness plan.

## Background

In recent years, the Amhara people living within and outside the region are being displaced due to politically provoked ethnic-based attacks. On December 14/2021, the Amhara Public Health Institute and the Regional Disaster Prevention and Food Security Commission reported that over 2,356,587 ethnically Amhara individuals had been intentionally uprooted from their indigenous residency in different regions and lived in 37 collective sites and the host communities (1). The cause of displacement was ethnic-based attacks against civilians, armed conflict, and escalating tensions in Benishangul-Gumuz and Oromia regions, and because of the war with Tigrayans Invading Forces in the northern part of the country.

Between October 2020 and June 25/2021, a total of 79,041 ethnically Amhara/Agew individuals had been displaced from Metekel zone of Benishangul Gumuz region because of ethnic-based attacks and lived in Ranch collective site, Chagni town. To facilitate emergency humanitarian responses, the Amhara regional government established Emergency Coordination Center (ECC) in Chagni. Following this, the Amhara Public Health Institute prepared a health and nutrition emergency response plan and activated an onsite Public Health Emergency Operation Center (PHEOC) in Chagni town on January 11/2021. Thus, the overall health and nutrition emergency responses in Ranch/Chagni collective site were delivered through an onsite PHEOC team in collaboration with the ECC and other humanitarian actors.

In developing countries like Ethiopia, the health needs of IDPs are not well-known and are poorly met in these countries (2). To overcome these, we established an onsite PHEOC to ensure that the health and nutrition needs of the IDPs are met and delivered according to international humanitarian response standards like Sphere Handbook, with a coordinated effort.

Despite the large numbers of IDPs/refugees in different countries, documented experiences about health service delivery approaches and methodologies in the camp are limited and previous studies are narrowed on the health needs of the IDPs, or a single disease/service and the accessibility of the services, but the way how we deliver humanitarian services is forgotten. In this paper, we have tried to report a more comprehensive picture of humanitarian operations in the Ranch camp with a more emphasis on the process and service delivery approaches to fill the above literature gap.

## Emergency response methodology

### Response design and period

On January 01/2021, the Amhara Public Health Institute activated the regional offsite Public Health Emergency Operation Center (PHEOC) in Bahir Dar city. Similarly, on January 08/2021, the regional government established Emergency Coordination Center (ECC) in Chagni town. Following this, the institute also established an onsite PHEOC in Chagni town on January 11/2021. Humanitarian responses were officially started in November 2020 and ended on June 25/2021 after 8 months of operations. Cessation of health service delivery was carried out once the IDPs were fully returned to their place of origin.

### Onsite PHEOC/IMS organogram

**Figure 1:**
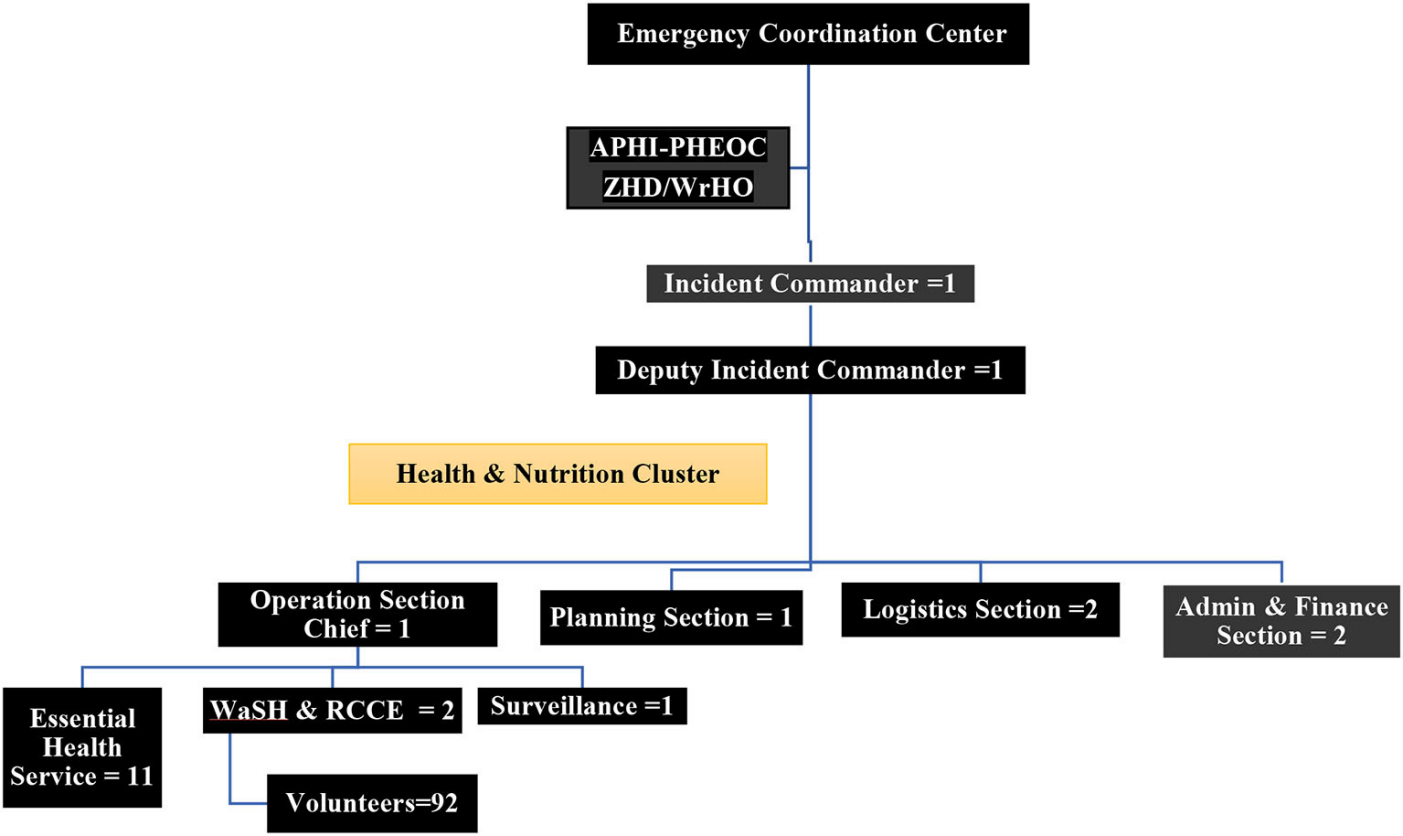
Ranch collective site onsite-PHEOC incident management structure/organogram, November 2020 to June 2021, Changi, Ethiopia.

The Incident Management Structure (IMS) for Ranch EOC was adapted from the World Health Organization handbook for developing a PHEOC (3). As shown in Figure 1 below, the onsite PHEOC has its incident commander, deputy incident commander and operation section chief and unit members. The onsite PHEOC is directly accountable to the ECC in Chagni town and reports to the offsite PHEOC in Bahir Dar city. Besides, the onsite PHEOC had a direct line of communication with the Awi Zonal Health Department, Chagni Town Health Office, and other humanitarian actors.

### Human resources recruitment and staffing

A total of 18 healthcare workers (HCWs), 4 drivers and 92 volunteer IDPs were staffed in the IMS structure. We mobilized the PHEOC team from Amhara Public Health Institute (3), Awi Zonal Health Department (1), Chagni Town Health Office (1), Chagni Primary Hospital (4), Chagni Health Center (1), MSF Spain (8), non-employed volunteer HCWs (4) and volunteers IDPs (92). Volunteers were selected together with tent leaders using flexible criteria like having a grade 10 level of education, being a 24-h tent-resident, being among the displaced community, good communication skills with Amharic and Agewugna languages, and from each sex, tent, or kebele/district of displacement. Also, efforts had been made to recruit dedicated, passionate, and multi-disciplinary HCWs and drivers. All of these were staffed in the IMS organogram of Ranch collective site as *full-time workers* (Figure 1).

Despite this, there was no mobile health and nutrition team deployed in the camp. However, a different surge team was deployed for a short period in an *ad-hoc* manner. The first was a team of 7 mental health and psychosocial support (MHPSS) professionals deployed by the Ethiopian Public Health Institute for 21 days. The second was a team of 5 general practitioners deployed by Amhara Emergency Fund for 12 days, and the third was a team of 20 multi-disciplinary medical experts deployed by Fewus Charity Association for 5 days and the last was a team of 24 MHPSS experts deployed for 20 days by the Amhara Public Health Institute in collaboration with Injibara University and Awi Zonal Health Department.

### Site selection, setting and population

**Figure 2:**
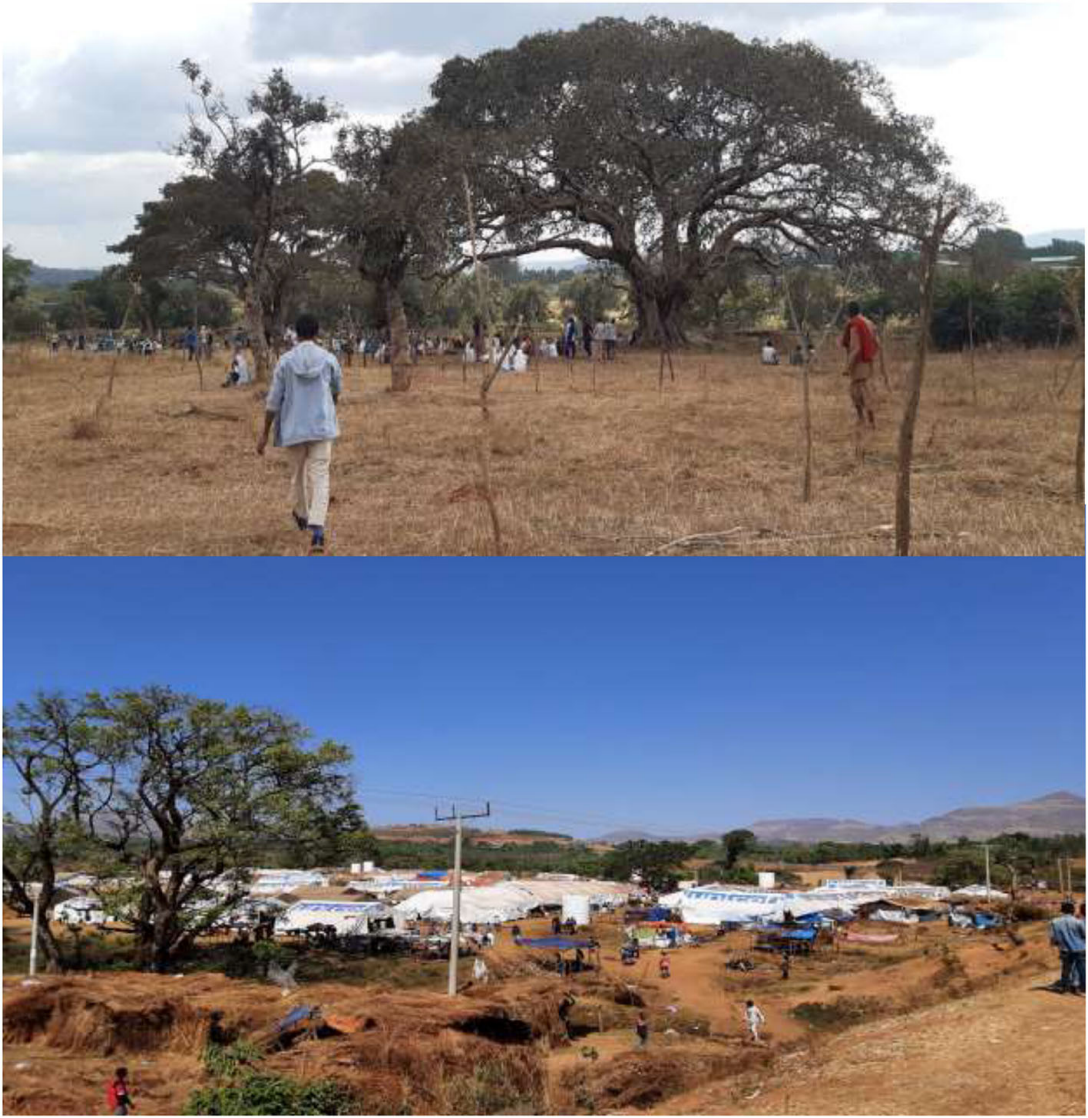
Ranch self and planned settlement site, Changi, Ethiopia, November 2020 up to June 2021.

Initially, the IDPs resided in an open-field self-settlement site called “Ranch,” which is part of Chagni town, Amhara region. They were shaded in an extremely large tree called *Sycamore*, meaning “*Warka*” in Amharic (Figure 2). Then after, in the first week of January, ECC team was deployed in Chagni town. Immediately, the team had made a discussion with Chagni town and Gangua district administrative bodies about the humanitarian response and jointly selected an appropriate settlement site called “Ranch collective site or IDPs site,” which is located nearly 4 kilometers from Chagni town (Figure 2). Also, it is 506 Km far from Addis Ababa, the capital city of Ethiopia in the northwest direction and 177 km from Bahir Dar, the capital city of Amhara regional state.

Out of 79,041 IDPs, 31,868 (40.3%) of them had lived in the camp and the rest 47,173 IDPs were living in the host community while collecting their humanitarian aids like food and non-food items, and free medical services from Ranch IDPs site. They were displaced from seven districts of Metekel zone of Benishangul-Gumuz region: namely, Dangur, Dibatie, Mandura, Bullen, Guba, Gilgel Beles and Wombera. But the majority were from Dangur (40.6%) and Dibatie (21.8%) districts. In the camp, there was a registry of 1,448 children under five, 141 pregnant and 402 lactating women. An additional 313 pregnant women who were living in the host community had received essential health services from the camp. There was also a non-registered/uncounted number of children under five who were living in the host community while receiving health services from the camp.

### Partners involved in health and nutrition emergency response

Four non-governmental organizations had been engaged in health and nutrition emergency response at Ranch collective site. The MSF Spain was the leading humanitarian agency acting on hygiene and sanitation issues, risk communication and community engagements (RCCE), provision of drug and medical supplies, and essential health service delivery. Also, UNICEF had been acting on nutrition emergencies, provision of emergency drug kits and financial support. The Catholic Relief Services (CRS) was supplying water for the IDPs and supporting hygiene and sanitation-related activities. Johns Hopkins Center for Communication Programs supported risk communication and community engagement activities focusing on COVID-19 and Gender-Based Violence. All agencies were providing technical support for the PHEOC and ECC teams. They were reporting their daily activities to the PHEOC planning section.

### Emergency response monitoring and evaluation

We had prepared a three-month health and nutrition emergency response plan. From this plan, we prepared weekly and daily Incident Action Plans (IAP) throughout our stay. We also evaluated our emergency response status on a daily and weekly basis with the PHEOC and ECC teams. Besides, the offsite PHEOC which is situated in Bahir Dar was closely following the onsite response on a daily and weekly basis with acting partners. Also, we had been conducting weekly cluster meetings with health and nutrition sector humanitarian actors. Throughout the response period, we produced and distributed 33 humanitarian response Situation Reports (SitRep). Our means of communication during the response were through a phone call, telegram, Facebook, email and paper-based. Daily onsite work attendance and meeting minutes had been recorded throughout the response period. Finally, an after-action review meeting had been conducted and all contributing agencies and individuals were acknowledged and certified by the Amhara Public Health Institute on July 10/2021 at Debre Tabor town. The success of the onsite PHEOC was evaluated based on the accessibility/availability of 24-h essential health/nutrition services, the ratio and adequacy of WaSH indicators to the displaced persons, the existence of a vibrant surveillance system in the camp, an appropriate risk communication strategy and other national/international humanitarian standards.

### Data reporting and information management

Throughout the response period, data were collected daily using a structured data collection and reporting tool. The tool addressed pertinent indicators regarding essential health services, WaSH and RCCE activities, and other health-related services. The primary data sources were clinical service registries, direct observations, the IDPs, daily situation reports, and other records. All humanitarian partners were also reported to the onsite PHEOC planning section on daily basis. Two of the investigators were leading the overall health and nutrition emergency response throughout the camp life. Thus, they were in charge of coordinating the data collection and reporting activities on daily basis as part of their duty for a prompt response. Data quality was assured through daily data cleaning, discussion with the PHEOC teams, training, and supervision. Daily, data entry and analysis had been done using Microsoft Excel 2016. Situation updates had been released regularly for concerned bodies.

### Definitions of terms

**Internally displaced persons:** are individuals who had been forced or obliged to flee from their homes or places of habitual residence due to natural or man-made disasters, and who have not crossed an internationally recognized state border (4, 5).

**A public health emergency operation center:** is a place within which the preparation, response, and recovery phases of public health emergencies are better coordinated. Sometimes, in the document, we used the term EOC to represent PHEOC (6, 7).

**On-site PHEOC:** is a tentative PHEOC established by the Amhara Public Health Institute in Chagni town, Ranch IDPs site, that served as a hub for better coordination of health and nutrition emergency response.

**The incident management system (IMS):** It is an emergency management structure with protocols and procedures that provides an approach for the coordination of response through a PHEOC, primarily to respond to and mitigate the effects of all types of emergencies (6, 7).

**Emergency coordination center (ECC):** is operationally defined as a multi-sectoral team established by the regional government to carry out all the five functions of the emergency operation center in Chagni town, Ranch IDPs site.

**Situation report (SitRep):** A routinely produced report that provides current information about health and nutrition emergency response at Ranch IDP site, its major gaps/challenges, and the way forward.

**Public health emergency:** this means an extraordinary event that constitutes a public health risk through the spread of disease and potentially requires an immediate and coordinated national or international response (3).

**Diagnosis of cases:** Malaria, diabetes mellitus and pregnancy were diagnosed using a rapid diagnostic test kit, random blood sugar test, and urine test strips, respectively.

## Results

### Essential health services

#### Temporary clinic set up

Initially, essential health services had been delivered at Chagni Primary Hospital free of charge. After a month, a “Temporary Clinic” at Ranch collective site was established. Yet, free medical services had been delivered at nearby hospitals through strong referral linkages.

**Figure 3:**
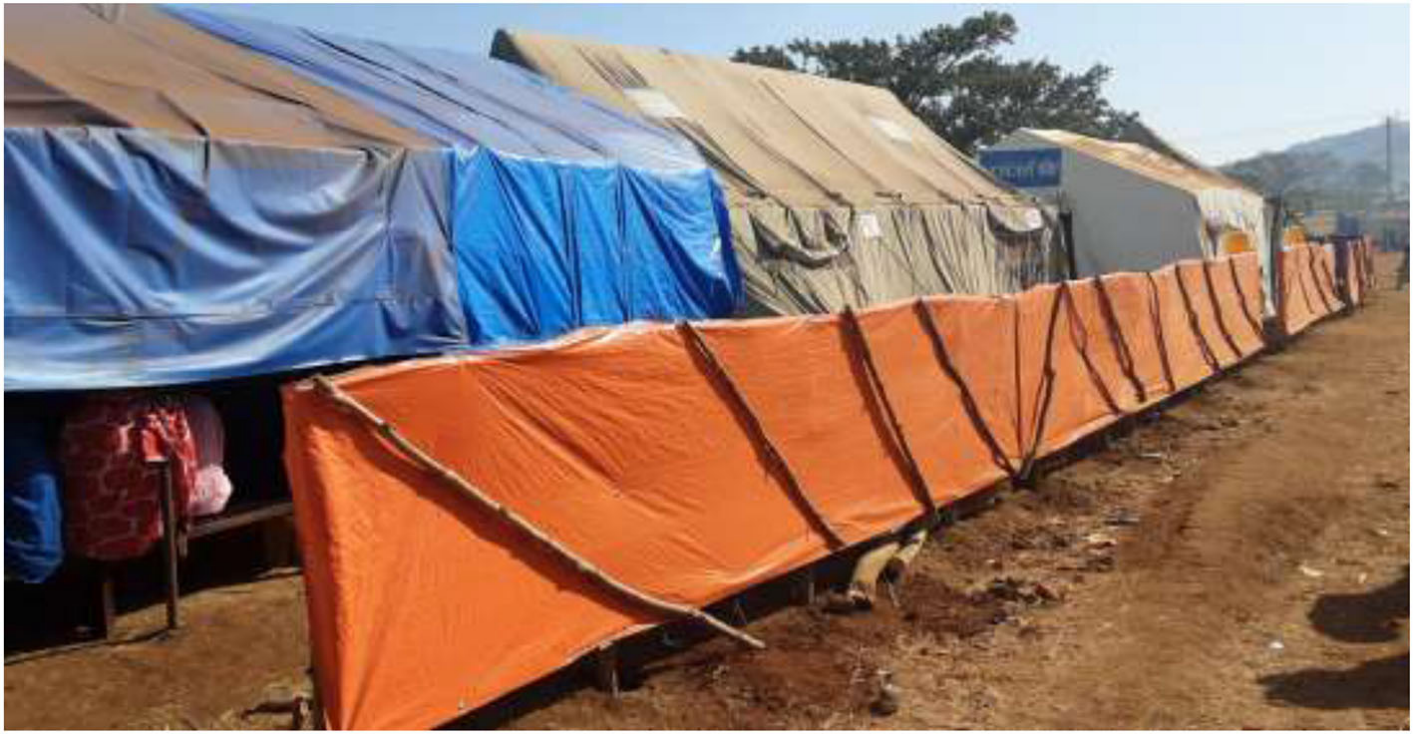
Layout of Ranch temporary clinic, Changi, Ethiopia, November 2020 up to June 2021.

The Ranch temporary clinic was established with five UNICEF-funded tents, having nine service delivery outlets: namely, two adult outpatient departments (OPDs), two under five OPDs, one dispensary, one maternal and child health service room, one mental health and psychosocial support room, waiting and triage rooms (Figure 3). Eleven healthcare workers (HCWs) were assigned to the temporary clinic; 3 were from Chagni primary hospital, 4 were volunteer health professionals and the rest 4 were MSF Spain staff. MSF Spain also assigned cleaners and guards to the clinic. The APHI allocated an ambulance to patient referral that also provides transportation services for clinicians. Emergency nutrition and mental health services were also integrated with other clinical services in the clinic. MSF Spain offered a myriad of supports like human resources, and the provision of drugs and medical supplies. Simple and rapid laboratory testing options like malaria Rapid Test Kits, pregnancy test strips, Hemoglobin, Random Blood Sugar, and urine dipstick were availed in the temporary clinic.

#### Essential health services

During the camp life, a total of 33,410 IDPs accessed essential health services from the Ranch temporary clinic and Chagni primary hospital through referral. Among these, 31,832 (95%) were cases with a kind of illness that needs medical interventions, while the rest had come for health promotion and disease prevention services. The average outpatient visit per day was 200 (150–250) cases. Regarding the age of patients, 1,938 (6.1%) were children <1 year, 5,381 (16.9%) aged one up to 4 years, 4,490 (14.1%) in between 5 and 14 years and the rest majority 20,022 (62.9%) were adults of 15 years and above. Mental health and psychosocial support had been given at least for 1,803 cases. Furthermore, specialized medical services like trachomatous trichiasis surgery (for 30 patients), cataract surgery (for 8 patients) and other minor and major surgical services (such as 13 caesarian sections) had been done through an outreach and referral system. In addition, a sputum sample for tuberculosis (TB) was collected from 120 suspected cases and 1 pulmonary TB case was detected and 5 contacts were screened negative. There was a total of 4 adult deaths among the IDPs. The cause of death was car accidents on the road, gun shoots and present chronic illness.

#### Maternal and child health services

Four hundred fifty-four pregnant women had received Antenatal Care (ANC) services at least once and 137 of them gave birth at health facilities, including 13 cesarean sections. The rest, 317 (69.8%) women, were returned to their original residencies while they were pregnant. Post-natal care services were delivered to 137 women. Ultrasound investigation had been done for 73 pregnant women during their ANC follow-up. Besides, 542 mothers received modern contraceptive methods. Childhood vaccinations had been given on a routine and campaign basis. In general, 137 children received the first dose of oral polio vaccine, 155 children got pentavalent 1st dose, 112 took pentavalent 2nd dose, 92 pentavalent 3rd doses, 74 and 66 children received the first and second dose of measles, respectively. Besides, 837 children under five had got supplementary measles doses through the campaign and 1,280 adults received the first dose of AstraZeneca's COVID-19 vaccine. Also, 91 adolescent girls aged 14 years got the human papillomavirus vaccine through a campaign. We vaccinated all eligible children living in the camp. Most children were fully vaccinated before displacement. That is why the number of vaccinated children in the camp seems low. Yet, few children that were living outside the camp might remain unvaccinated.

#### Emergency nutrition services

A total of 1,448 children under five had been screened monthly. Of these, 46 (3.2%) were severely malnourished, and 75 (5.2%) were moderately malnourished with a Global Acute Malnutrition rate of 8.3%. Forty-one severely malnourished children (89.1%) were linked to outpatient therapeutic feeding and/or stabilization centers. On the other hand, 141 pregnant and 402 lactating women had been screened regularly and 135 (25%) were found to be moderately malnourished. The rest pregnant women were living in the host community, and we failed to screen them on monthly basis; but MUAC was taken irregularly. For mothers and children who were affected with moderate malnutrition, additional fortified foods had been given monthly. During the camp life, each household had got a general food ration of 15 kilo grams per person per month. An additional food commodity had been given regularly to households with children under-five, pregnant/lactating women and people living with HIV, to prevent acute malnutrition.

### Water, hygiene and sanitation (WaSH)

**Figure 4:**
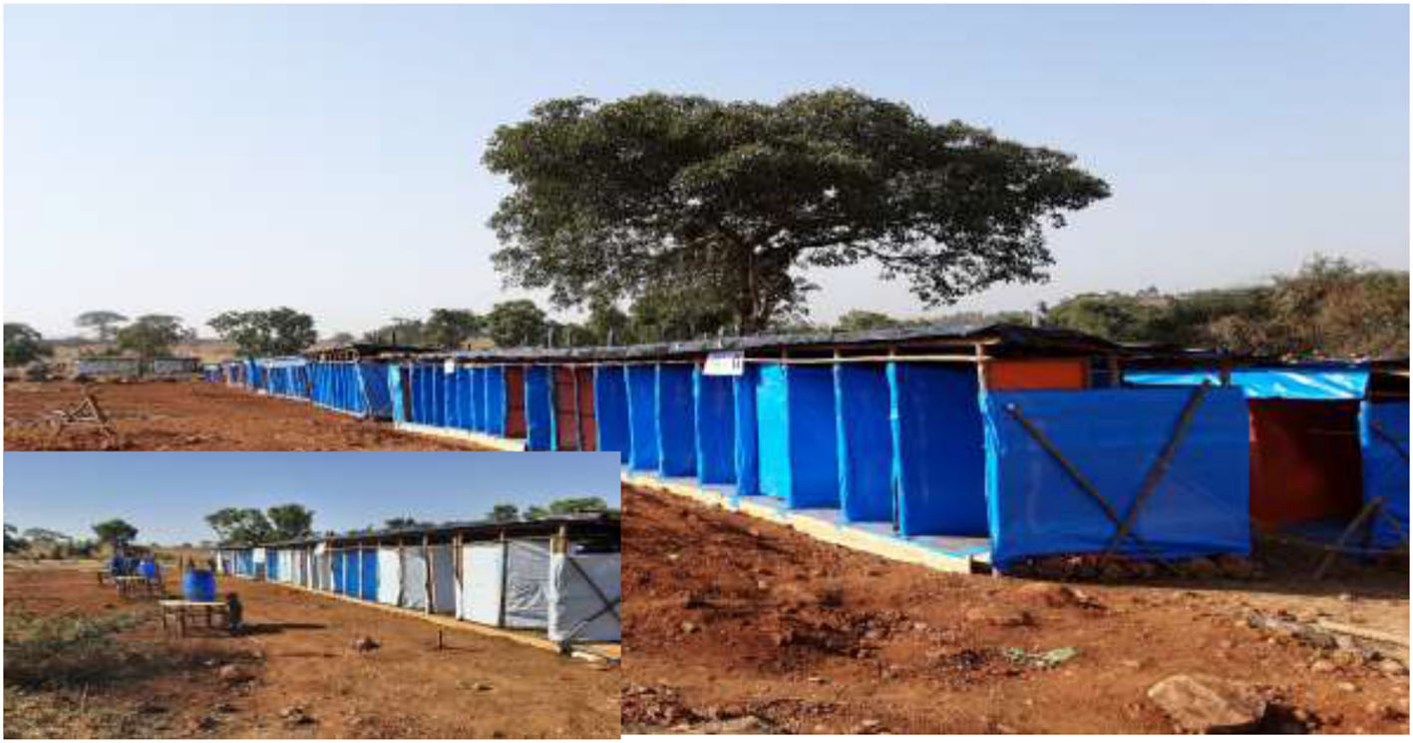
Trench latrine seats constructed in Ranch collective site, Changi, Ethiopia, November 2020 up to June 2021 (*N* = 194).

A total of 194 trench latrine seats (100 for females), 74 shower rooms (50 for females) and 50 hand washing facilities were constructed (Figure 4). One hundred forty-four of the latrine seats and all the shower rooms were constructed by MSF Spain, while the rest 50 latrine seats were built by the Catholic Relief Services. All toilets were sited appropriately and adequately distanced from any surface or groundwater source. The estimated distance between dwellings/shelters and shared toilets ranged from 60 to 100 meters. All toilets have internal locks, easy to use and keep clean, but no lighting. Besides, they were not suitable for fly and mosquito breeding, had a minimal smell and had adequate space for different uses, which allows for the dignified cleaning, drying and disposal of women's menstrual. Further, toilets were provided with easy access to water for handwashing, anal cleansing, and flushing.

**Figure 5:**
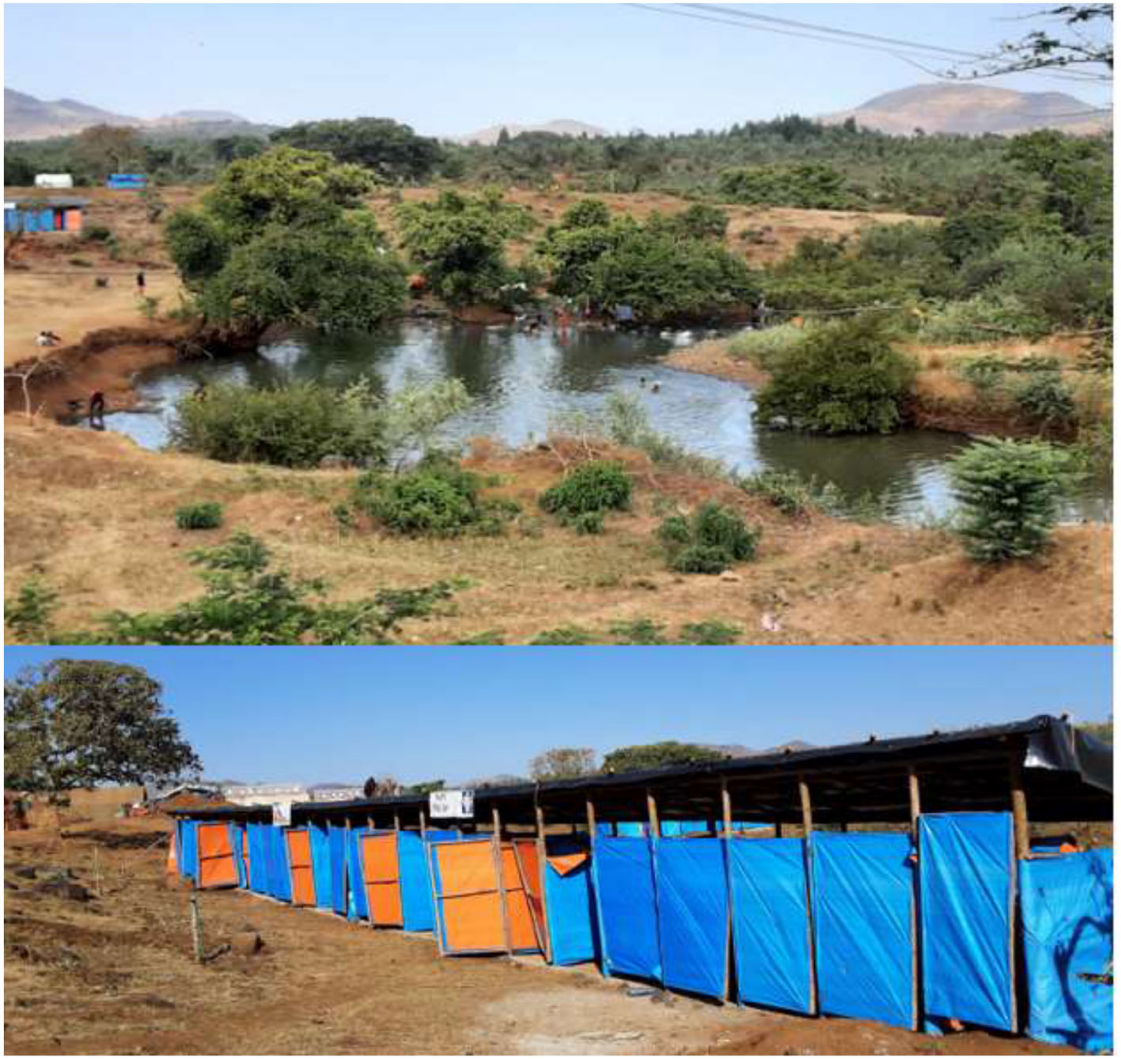
Shower rooms and swimming pool in Ranch collective site, Changi, Ethiopia, November 2020 up to June 2021 (*N* = 194).

Two water tracking rotos with a capacity of 10,000 liters were installed for water supplies to latrines and shower rooms. In addition to the shower rooms, there is a naturally occurring body of water, which serves as a recreational area; especially male IDPs were taking a shower in the pond and river while recreating themselves (Figure 5). At least 2 soaps per person per month had been provided for bathing and laundry purpose. Women's dignity materials were also distributed regularly to women of reproductive age groups. Moreover, 19 medium-sized solid waste management pits have been prepared around the settlements. Thus, all tents have access to designated communal solid waste collection points. The WaSH team and the IDPs had conducted regular sanitation campaigns. Also, tent inspection has been conducted routinely by trained IDP volunteers. Generally, there were no human feces present nor solid wastes accumulated around the shelters or settlements. And we judged that the WaSH services in the camp were adequate, appropriate, and acceptable to the IDPs.

### Risk communication and community engagement

We established Mini Media with support from Johns Hopkins Center for Communication Programs through its “Communication for Health” program, which serves as a broadcasting center. In addition, we endorsed a tent-to-tent risk communication approach to address residents in each tent. Thus, health information on priority health issues and treats had been effectively delivered routinely. The main areas of risk communications were environmental and personal hygiene, mental health problems, COVID-19, gender-based violence, scabies, tuberculosis, sexually transmitted disease, community/event-based surveillance, water and food-borne diseases, malnutrition, malaria and extra. That is how we empowered the IDPs and engaged them in emergency WaSH/IPC implementation, event-based surveillance, essential health service uptake, and other humanitarian operations.

### Public health surveillance

In the camp, both indicator and event-based surveillance approaches had been phased for early detection and prompt response to public health threats. Rumors and early warning signals have been verified soon through active surveillance. For instance, one sample from cholera and two samples from measles suspected cases were taken to the regional laboratory and all were tested negative. Standard surveillance tools like data collection and reporting, case definitions and guidelines had been availed and orientation training for the PHEOC team had been given. Data on clinical case management had been collected and analyzed on daily basis and disseminated through situation reports.

Throughout the camp life, a total of 31,832 (95% of the total OPD visits) IDPs had been medically treated with a kind of acute or chronic medical illness (Table 1). The most diagnosed health problems were pneumonia (13.2%), acute febrile illness (11.1%), intestinal parasite (9.5%), acute upper respiratory tract illness (7.7%), none-bloody diarrhea (6.8%), dyspepsia (6.2%) and ophthalmologic problems (5.8%). Scabies and malaria (RDT confirmed) contribute 4.6% and 2.4% of the cases. Epidemiological trends of common epidemic-prone diseases like malaria, scabies and diarrhea were observed closely and found to be constant throughout the response period. There was no occurrence of case build-up or outbreaks of epidemic-prone diseases.

**Table 1:** Total number of morbidities diagnosed in Ranch collective site, Chagni, northwest Ethiopia, November 2020 to June 2021 (*N* = 31,832).

**Disease type**	** <1 year**	**1–4 year**	**5–14 year**	**≥15 years**	**Total**	**Proportion**
Pneumonia	368	837	536	2,444	4,185	13.1
Acute febrile illness	113	567	467	2,393	3,539	11.1
Intestinal parasite	242	564	854	1,363	3,023	9.5
Acute upper respiratory tract infection	458	902	426	655	2,442	7.7
Diarrhea (none bloody)	331	713	308	808	2,160	6.8
Dyspepsia	0	0	4	1,985	1,989	6.2
Ophthalmologic cases	111	383	447	915	1,856	5.8
Rheumatoid arthritis	0	60	7	1,650	1,717	5.4
Others medical illness	33	146	320	1,167	1,667	5.2
Mental health and psychosocial support	0	24	185	1,486	1,695	5.3
Scabies	124	433	199	698	1,454	4.6
Urinary tract infection	0	27	127	1,036	1,191	3.7
Bloody diarrhea	67	298	245	423	1,034	3.2
Dermatologic cases	28	77	125	689	918	2.9
Malaria (RDT confirmed)	38	177	111	441	766	2.4
Injury/accident, trauma	7	37	54	328	425	1.3
Migraine headache	0	0	6	398	404	1.3
Hypertension	0	0	0	304	304	1.0
Minor surgery	5	14	27	182	227	0.7
Wound Infection	4	11	21	141	176	0.6
Asthma	0	0	8	146	154	0.5
Sexually transmitted infections	0	0	0	138	138	0.4
Known HIV cases	0	0	4	104	108	0.3
Malnutrition U5 children (SAM and MAM)	10	111	0	0	121	0.4
Diabetes mellitus	0	0	2	83	85	0.3
Presumptive tuberculosis	0	0	7	42	49	0.2
Chronic liver disease	0	0	0	4	4	0.0
Total	1,938	5,381	4,490	20,022	31,832	100

## Discussion

The overall health and nutrition emergency response in the Ranch collective site was coordinated and delivered by the Ranch onsite PHEOC which was established in Chagni town. The primary aim of this coordination platform was to ensure that health and nutrition emergency responses are delivered according to national and international humanitarian response standards (8, 9). We structured the onsite PHEOC to carry out all the five-basic functions of EOC. The incident commander and/or the deputy had played basic managerial functions like organizing/assembling the PHEOC team, providing onsite training, preparing terms of reference, overseeing the day-to-day operation of the PHEOC, conducting discussions with state and non-state actors and receiving a command from the higher levels. This onsite coordination platform enabled us to deliver quality health and nutrition services for all groups of IDPs, as written in the emergency response plan. Besides, it allowed us to early understand public health needs and problems, response constraints and challenges in the camp.

Overall, 33,410 IDPs received essential health services from the clinic. However, despite a high incidence of epidemic-prone diseases in similar settings (10), there was no occurrence of an outbreak of measles, cholera, polio, meningitis, and pertussis in the Ranch camp. The averages daily outpatient visits in Ranch temporary clinic were higher than what most primary hospitals report in the Amhara region. These much health services had been delivered with few, but firmly committed health professionals. Also, specialized medical services had been delivered on an outreach basis, as well as through a referral system at the nearest hospitals. For instance, trachomatous trichiasis and cataract surgeries had been done for 30 and 8 ophthalmic patients, respectively. Not only this, sputum samples for tuberculosis (TB) had been collected from 120 presumptive TB cases and one pulmonary TB case was detected and 5 contacts were also screened negative. In addition, mental health services had been integrated within the clinic and delivered according to the WHO recommendation for mental health services in emergency settings (11). In this regard, the PHEOC team believes that health services had been delivered based on their needs and according to national and international standards and protocols (4, 9, 11–13). Also, a vibrant emergency logistics system had been in place that delivered an ample amount of emergency drugs and medical supplies at the right time for Ranch temporary clinic and Chagni hospital.

Under the WaSH operation section unit, a total of 194 trench latrine seats, 74 shower rooms and 50 hand washing facilities had been constructed at the Ranch IDP site. Although having an adequate number of WaSH facilities by itself was not a guarantee to keep the camp and environment free from human feces and solid wastes. Hence, conducting a regular compound sanitation campaign was mandatory to keep clean the settlement from any solid wastes. As well, latrine seats had been washed on daily bases through the coordinated efforts of community volunteers. The main challenge in this regard was inadequate water supply to wash latrines on daily bases. Interestingly, river water was available within a 50–70-meter distance and we fetched from it.

Even though the ratio of latrine seats to that of the IDPs was not comparable with the Sphere standards, the other elements of WaSH were as per the recommendation in Sphere Handbook (9). For instance, waiting time for the toilet was zero, all latrine seats were within acceptable distances from shelters, they had internal locks, easy to use and keep clean. As well, they were not suitable for fly and mosquito breeding, had minimal smell and had adequate space for different uses, which allowed for the dignified cleaning, drying and disposal of women's menstrual. Largely, there were no human feces present nor solid wastes accumulated around the shelters or settlements. In this regard, volunteer IDPs had a substantial contribution to creating clean and safe settlements to the extent of being role models for the surrounding residents.

As a whole, the major response challenges were shortage of health professionals to fulfill the IMS structure, scarcity/inflexibility of budget, shortage of ambulances, inadequate water supply for latrine and shower rooms, and limited experience in collective center management and humanitarian services. A study conducted in three Sub-Saharan African countries also revealed that insufficient knowledge and experience about the health care needs of displaced populations was a challenge in Somalia, Kenya and Ethiopia (2). Also, another study from Somalia reported that shortage of human resources and insufficient funding were among the major factors influencing humanitarian services (14). Besides, the roles and responsibilities of the region, zone, district, and facility were not clearly stated, understood, and agreed upon by the respective organizations. Hence, some governmental organizations were not happy to take responsibility for the emergency response, considering this humanitarian response as a regional responsibility. The negative effects of such misconception were resolved and/or minimized by engaging all levels of the health sector in the onsite PHEOC. Also, frequent discussions had been held with the respective organizational leaders regarding the humanitarian response and its challenges.

For future similar emergencies that need the coordinated efforts of all levels of the health sector, joint discussion by inter and intra-sectoral leaders should be held about the shared and/or individual roles and responsibilities in the emergency response. Perhaps, this kind of discussion would be more fruitful and valuable if it is conducted at the initial phase of the emergency with a binding agreement by top-level managers and each coordination level should be accountable as stated in the agreement. Another lesson we took is the importance of having a pool of health workers who are experienced in EOC and humanitarian operations that are readily available for anytime deployments in the region. Besides, we have to develop/customize standards for humanitarian response (WaSH, surveillance, RCCE, temporary clinic, etc), develop health and nutrition emergency response guidelines, training materials, terms of reference for onsite EOC, and develop a training curriculum entitled *Leading in Emergencies* for emergency leaders. Above all, there should be directives regarding financial, human and material resource mobilizations and utilization during emergency responses. This directive should answer the basic concept of EOC and its unique future to bypass any bureaucratic government structure that hinders or delays timely response, and all government bodies and humanitarian actors should legalize and deeply understand it. Another powerful lesson we took is the need to have pre-existing buildings and infrastructures that serve as a multi-purpose collective center. These “collective centers” can be built in selected areas of the region with easily modifiable rooms/designs to use for similar purposes like isolation/quarantine centers, epidemic treatment centers, warehouses, etc.

## Conclusion and lessons learned

We adequately met the health and nutrition needs of the IDPs, as stated in the Sphere humanitarian handbook. We have got so many lessons from the emergency response. The most pertinent lessons are the importance of a strong Incident Management System and onsite coordination platforms like PHEOC in managing complex emergencies, the need to develop a training curriculum entitled *Leading in Emergencies* for key PHEM leaders, building multi-purpose collective centers with infrastructures, having a roster of experienced experts on humanitarian operations, develop humanitarian response standards, guidelines, TOR, training materials, directives and PHEOC legal framework regarding financial, human and material resource mobilizations and utilization during emergencies and risk/vulnerability driven preparedness plan are among the key determinants of effective response.

## Data availability statement

The raw data supporting the conclusions of this article will be made available by the authors, without undue reservation.

## Ethics statement

The Amhara Public Health Institute Ethical Review Committee has approved this study. Administrative permission to access and publish the data was obtained from the Amhara Public Health Institute administrative bodies. Written informed consent for participation was not required for this study.

## Author contributions

TA, BB, and AA conceived and designed the response method, which was needed to establish an Offsite and Onsite PHEOC and they supervised and monitored the entire process of the emergency response. MA, BB, and AA were Offsite PHEOC Incident Response Leader, Incident Manager, and Deputy Incident Manager, respectively. TA and EH were Onsite PHEOC Incident Commander and Deputy Incident Commander respectively, and they were directly involved in humanitarian responses in the camp, daily operations, day-to-day data collection, response team assembly and overall coordination of the team in the camp. TA analyzed the data and wrote the first draft of the manuscript. BB and AA reviewed and modified the drafts of the manuscript. MH took part in the whole process of the response and reviewed and modified the drafts of the manuscript. MA also participated in the design, supervised, and monitored the whole process of the emergency response. All authors have read and approved the different study steps and the final version of the manuscript.

## Conflict of interest

The authors declare that the research was conducted in the absence of any commercial or financial relationships that could be construed as a potential conflict of interest.

## Publisher's note

All claims expressed in this article are solely those of the authors and do not necessarily represent those of their affiliated organizations, or those of the publisher, the editors and the reviewers. Any product that may be evaluated in this article, or claim that may be made by its manufacturer, is not guaranteed or endorsed by the publisher.

## Author disclaimer

The findings and conclusions are those of the authors and do not necessarily represent the views of the Amhara Public Health Institute/Regional Health Bureau.

## Abbreviations

APHI: Amhara Public Health Institute
ECC: Emergency Coordination Center
EOC: Emergency Operation Center
GBV: Gender-Based Violence
MHPSS: Mental Health and Psychosocial Support
HCWs: Health Care Workers
IDPs: Internally Displaced Peoples
IMS: Incident Management Structure
PHEOC: Public Health Emergency Operation Center
RCCE: Risk communication and community engagement
WaSH, Water: Sanitation and Hygiene.
